# Laparoscopic Removal of a Vaginal Foreign Body (Aerosol Cap): A Case Report

**DOI:** 10.7759/cureus.96241

**Published:** 2025-11-06

**Authors:** Nese Hayirlioglu, Hakan Guraslan, Merve Cumaoglu, Rabia Bozkurt, Ozlem Karabay Akgul

**Affiliations:** 1 Obstetrics and Gynecology, Bagcilar Training and Research Hospital, Istanbul, TUR

**Keywords:** case reports, foreign bodies, laparoscopy, minimally invasive surgical procedures, vaginal stenosis

## Abstract

Vaginal foreign bodies, although uncommon, may cause significant complications when retained for prolonged periods. Firm objects can lead to chronic inflammation, fibrosis, and potential injury to adjacent organs, making removal technically challenging. A 19-year-old woman presented with persistent foul-smelling vaginal discharge and spotting that had continued for approximately four years after she was unable to remove a deodorant cap inserted vaginally. Examination revealed severe fibrotic narrowing and obliteration of the vaginal canal, and computed tomography demonstrated the foreign body localized in the vaginal vault. Due to the depth of localization and high risk of injury to surrounding structures, vaginal extraction was deemed unsafe. Laparoscopic surgery with posterior colpotomy was performed, and the foreign body was successfully removed without complications. The postoperative course was uneventful, and subsequent follow-up showed complete healing of inflammation, although severe vaginal stenosis persisted. This case highlights that removal of vaginal foreign bodies through the vaginal route, while generally preferred, may not always be feasible. In circumstances where the vaginal opening is compromised, the tissue is fibrotic, and the object is located deeply, laparoscopy provides a safe and effective alternative to prevent injury and preserve organ function.

## Introduction

Vaginal foreign body is a rare but clinically important condition that may cause persistent foul-smelling or bloody vaginal discharge and, if retained for long periods, can lead to chronic inflammation and fibrosis. The type of object varies by age group and intent; small plastic toys or tissue paper fragments are more frequent in children, whereas deodorant or aerosol caps are often reported in older adolescents, typically associated with sexual experimentation [1].

Diagnosis requires careful history taking and physical examination. In sexually active patients, speculum evaluation may aid visualization of the object and any associated mucosal injury. When the diagnosis is uncertain or complications are suspected, imaging methods such as ultrasound, X-ray, or computed tomography can help define localization and potential involvement of adjacent organs [2].

While most foreign bodies can be removed transvaginally under direct visualization, surgical intervention may be required when removal is unsafe or unsuccessful. This case highlights a laparoscopic approach for the removal of an aerosol cap in an adolescent patient, emphasizing its role as a minimally invasive and safe option in cases of severe vaginal stenosis or deep localization.

## Case presentation

A nulliparous 19-year-old female presented to the outpatient clinic with complaints of foul-smelling vaginal discharge and intermittent bleeding. She reported that she had inserted an aerosol canister vaginally for masturbation approximately four years earlier but had been unable to remove the cap. On pelvic examination, a foreign object was palpable with the fingertip at a depth of approximately 8 cm from the vaginal introitus; however, the object was not visible, and the vaginal canal was almost completely occluded by dense fibrotic tissue. The cervix was not visible.

**Figure 1:**
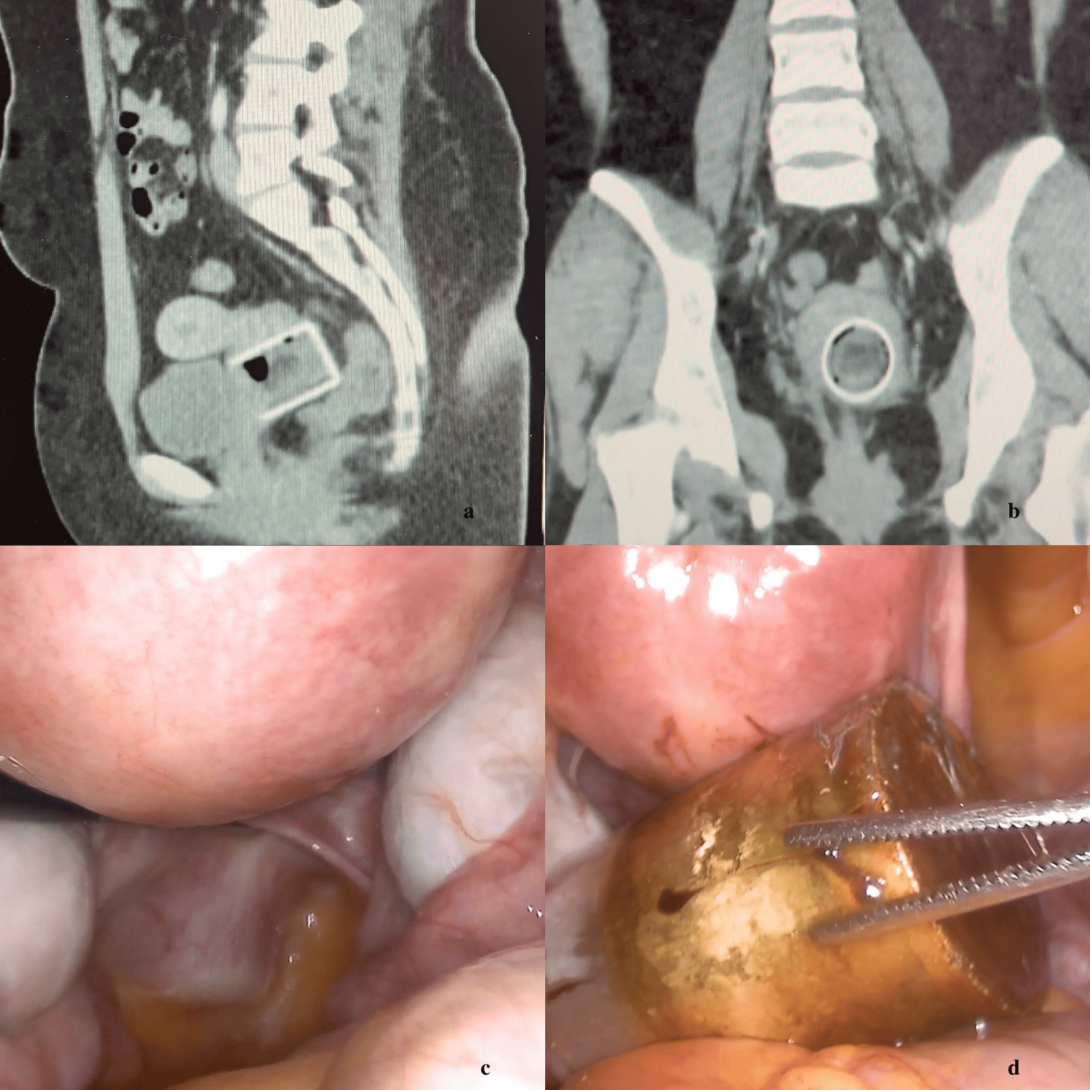
Images of foreign body a: Sagittal computed tomography image showing a metallic aerosol cap lodged in the vaginal vault posterior to the bladder. b: Axial computed tomography image of the foreign body c: Laparoscopic view demonstrating the foreign body reflection at the cervicovaginal junction. d: Removal of the cap through the posterior colpotomy incision.

A preoperative CT scan demonstrated a metallic aerosol cap measuring 35 mm × 55 mm, localized within the vaginal vault behind the bladder (Figure 1A, 1B).

Preoperative evaluation included complete blood count, C-reactive protein, and urine analysis, all within normal limits. Vaginal culture revealed Enterococcus faecalis growth, and metronidazole therapy was administered prior to surgery. During examination under anesthesia, the vaginal canal was found to be completely obliterated by fibrotic tissue, and the foreign body could not be safely visualized. To minimize the risk of bladder or rectal injury, the procedure was converted to a laparoscopic approach. The clinical stages, key findings, and corresponding procedures are summarized in Table 1.

**Table 1:** Summary of diagnostic, surgical, and postoperative findings in the present case This table summarizes the chronological progression of clinical findings, diagnostic investigations, surgical procedures, and postoperative outcomes in the reported case of retained vaginal foreign body (metallic aerosol cap). CBC: complete blood count; CRP: C-reactive protein; IV: intravenous.

Stage	Findings / Procedures	Details / Comments
Clinical presentation	Persistent foul-smelling vaginal discharge and spotting for 4 years	Patient reported prior insertion of a deodorant (aerosol) cap for sexual gratification.
Physical examination	Vaginal canal nearly occluded by dense fibrotic tissue	Foreign body palpable at ~8 cm depth; cervix not visible.
Imaging	CT pelvis (axial and sagittal, 3 mm slice thickness)	Metallic aerosol cap localized in the vaginal vault, posterior to the bladder.
Preoperative evaluation	Laboratory and infection screening	CBC and CRP within normal limits; vaginal culture grew Enterococcus faecalis; treated with metronidazole; prophylactic IV antibiotics administered before surgery.
Initial approach	Vaginal extraction under anesthesia attempted	Abandoned due to complete stenosis and poor visualization.
Definitive procedure	Laparoscopic posterior colpotomy	Aerosol cap removed safely without injury to adjacent organs.
Postoperative course	Uneventful recovery	Secondary vaginal procedure performed later to remove residual fibrosis.
Follow-up (3 months)	Complete resolution of inflammation	Persistent but asymptomatic stenosis; normal menstruation; no urinary or bowel symptoms; counseling provided for gradual dilation.

The operation began with vaginoscopy and cystoscopy, followed by laparoscopy. A clear image could not be obtained during vaginoscopy due to inflammation and narrowing. Cystoscopy revealed intact ureteric orifices and bladder trigone. Laparoscopically, a reflection of the metallic object was identified in the prerectal region near the cervicovaginal junction (Figure 1C). The rectum was gently mobilized to enter the rectovaginal space, and a posterior colpotomy was performed. The aerosol cap was dislodged by pushing it toward the abdomen with ovarian forceps inserted through the vagina (Figure 1D) and removed through the left lateral trocar site using an endo-bag (Video 1). Because the cap was metallic, fragmentation for transvaginal extraction was not feasible.

**Video 1:** Short video of the operation Laparoscopic removal of a vaginal foreign body (aerosol cap) through posterior colpotomy

**Figure 2:**
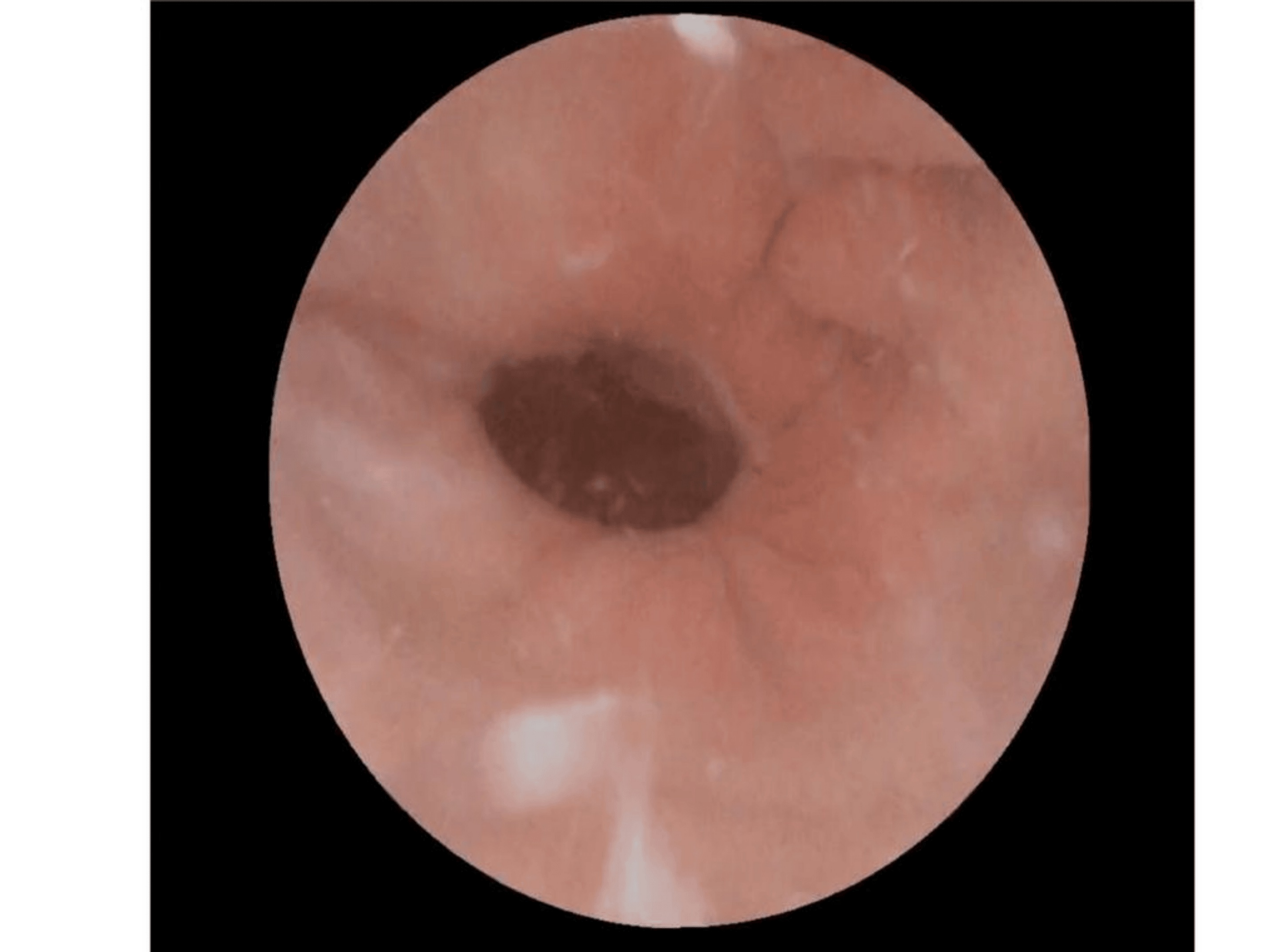
Vaginal stenosis Vaginoscopic image obtained three months postoperatively showing residual stenosis near the vaginal apex.

The procedure was uneventful. Six weeks postoperatively, inflammation had completely resolved. A secondary vaginal procedure was performed to excise residual fibrotic tissue, which had been deferred initially due to inflammation. At three-month follow-up, vaginoscopy showed persistent but stable stenosis near the vaginal apex (Figure 2). The patient reported normal menstruation and no urinary or bowel symptoms. Although sexual intercourse had not yet been attempted, counseling and gradual vaginal dilation therapy were recommended.

## Discussion

Vaginal foreign bodies, although uncommon, may lead to severe complications if retained for prolonged periods. The risk of injury appears to vary depending on the type and characteristics of the object [1].

In one review, 11 out of 40 reported cases of vaginal foreign bodies involved caps, nine of which were complicated by a genitourinary fistula, while none of the remaining 29 cases involving other objects such as plastic toys or batteries were associated with fistula formation, as shown in the supplementary data of that study [1]. This pattern suggests that aerosol caps, due to their shape, size, and rigid material, may exert higher pressure on adjacent tissues and therefore predispose to fistula formation. Consistent with this, aerosol caps have been repeatedly reported as the most common vaginal foreign bodies leading to acquired vesicovaginal fistulas [3,4]. This may be related to the distinctive mechanical effect of the cap’s open rim, which exerts concentrated edge pressure on adjacent tissues. Prolonged contact of this rigid rim can lead to progressive embedding into the vaginal wall, causing localized ischemia, mucosal laceration, and necrosis, which ultimately predispose to fistula formation.

Prolonged retention of vaginal aerosol caps has been frequently associated with the gradual development of urogenital fistulas, most likely due to sustained local pressure and chronic inflammatory changes. In such cases, patients often present with persistent urinary leakage as the initial and most troubling symptom, leading to delayed diagnosis and eventual identification of the underlying foreign body [5-7].

In addition, it should be noted that the process of foreign body removal itself may occasionally create a new urogenital defect, particularly when rigid objects such as aerosol caps are extracted transvaginally. The mechanical trauma induced during manipulation can disrupt previously intact tissues and result in a fistula that was not present before removal [3,4,8-10]. Furthermore, when a pre-existing defect is already present, instrumentation and removal maneuvers may exacerbate the condition by enlarging the fistulous tract, thereby complicating the clinical course and subsequent surgical management [11]. These reports highlight that such complications are not limited to simple bladder injuries, but may involve critical structures such as the bladder trigone and ureteric orifices [5,8,10,11].

In the present case, fibrosis and ischemic changes were localized immediately anterior to the sharp rim of the cap, which was oriented toward the posterior wall of the bladder, while the tissue posterior to the object remained intact. This spatial pattern of injury indicates that the open metallic rim exerted focal pressure and shear on the vesicovaginal septum, predisposing to localized necrosis and fibrosis. Given the metallic nature of the cap, fragmentation for transvaginal extraction was not feasible, and any attempt to remove it vaginally would have required traction across the injured area, with a high risk of bladder trigone damage or fistula formation. By contrast, the laparoscopic posterior colpotomy was performed through healthy vaginal apex tissue located behind the foreign body, allowing the cap to be gently mobilized and removed under direct visualization with minimal traction and without additional injury. This mechanism explains the biomechanical advantage of laparoscopy in preventing trauma during removal of rigid, embedded foreign bodies.

Another noteworthy aspect of this case is the unusually long delay before presentation. In cases involving vaginal foreign bodies, patients may postpone seeking medical attention due to feelings of embarrassment, shame, or fear of social stigma. This psychological barrier can contribute to a prolonged duration before diagnosis and treatment. In the present case, the patient’s hesitation to report her symptoms was most likely related to embarrassment, which explains the four-year delay in seeking medical care.

Depending on the intraoperative findings, foreign body removal may be performed vaginally or laparoscopically, as highlighted in more recent case reports [12]. However, in cases such as ours, where severe vaginal stenosis and deep localization of the object were present, the vaginal route posed significant risks. We therefore opted for direct laparoscopic removal. This approach allowed safe dissection, avoidance of injury to adjacent organs, and a minimally invasive postoperative course. Our case thus adds to the growing body of evidence that laparoscopy is not only a viable but sometimes preferable method for the management of high-risk vaginal foreign bodies, particularly aerosol caps.

## Conclusions

Removing vaginal foreign bodies through the vaginal canal is the most natural approach; however, in selected cases, the laparoscopic method may represent a safer alternative to prevent adjacent organ injuries and subsequent fistula formation. Our case demonstrates that when severe vaginal stenosis and deep localization of the object are present, laparoscopy offers both a safe and minimally invasive solution. Moreover, evidence from the literature indicates that aerosol caps, compared with other vaginal foreign bodies, are associated with a higher incidence of serious complications such as vesicovaginal and ureterovaginal fistulas. Therefore, in similar cases, the laparoscopic approach may represent a safe and minimally invasive alternative to reduce the risk of injury to adjacent organs and subsequent fistula formation.

## References

[1] Lehembre-Shiah E, Gomez-Lobo V (2024). Vaginal foreign bodies in the pediatric and adolescent age group: a review of current literature and discussion of best practices in diagnosis and management. J Pediatr Adolesc Gynecol.

[2] Anderson J, Paterek E (2023). Vaginal foreign body evaluation and treatment. StatPearls [Internet].

[3] Bansal A, Kumar M, Goel S, Aeron R (2016). Vesicovaginal fistula following insertion of a foreign body in the vagina for sexual gratification: could it be catastrophic?. BMJ Case Rep.

[4] Vilimas J, Baseviciene I, Kilda A, Puzinas A, Verkauskas G (2015). Vesicovaginal fistula in adolescent girls: incidence and management. J Pediatr Adolesc Gynecol.

[5] Bandarkar AN, Adeyiga AO, Shalaby-Rana E (2017). Ureterovaginal fistula secondary to retained vaginal foreign body in a young girl. Radiol Case Rep.

[6] Hanai T, Miyatake R, Kato Y, Iguchi M (2000). Vesicovaginal fistula due to a vaginal foreign body: a case report. Hinyokika Kiyo.

[7] Mengistu Z, Ayichew Z (2022). Large vesicovaginal fistula after vaginal insertion of a plastic cap healed with two weeks of catheterization: a case report. Int Med Case Rep J.

[8] Lo TS, Jaili SB, Ibrahim R, Kao CC, Uy-Patrimonio MC (2018). Ureterovaginal fistula: a complication of a vaginal foreign body. Taiwan J Obstet Gynecol.

[9] Fourie T, Ramphal S (2001). Aerosol caps and vesicovaginal fistulas. Int J Gynaecol Obstet.

[10] Binstock MA, Semrad N, Dubow L, Watring W (1990). Combined vesicovaginal-ureterovaginal fistulas associated with a vaginal foreign body. Obstet Gynecol.

[11] D'Elia C, Curti P, Cerruto MA, Monaco C, Artibani W (2015). Large urethro-vesico-vaginal fistula due to a vaginal foreign body in a 22-year-old woman: case report and literature review. Urol Int.

[12] Sharaf OM, Wilkinson EA, Elbadri E, Weber LeBrun EE (2023). Retention of a foreign body in the vagina of an adult for 13 years: a case report. Cureus.

